# A Community-Driven, Openly Accessible Molecular Pathway Integrating Knowledge on Malignant Pleural Mesothelioma

**DOI:** 10.3389/fonc.2022.849640

**Published:** 2022-04-26

**Authors:** Marvin Martens, Franziska Kreidl, Friederike Ehrhart, Didier Jean, Merlin Mei, Holly M. Mortensen, Alistair Nash, Penny Nymark, Chris T. Evelo, Ferdinando Cerciello

**Affiliations:** ^1^ Department of Bioinformatics - BiGCaT, NUTRIM, Maastricht University, Maastricht, Netherlands; ^2^ Department of Bioinformatics - BiGCaT, MHeNs, Maastricht University, Maastricht, Netherlands; ^3^ Centre de Recherche des Cordeliers, Inserm, Sorbonne Université, Université de Paris, Functional Genomics of Solid Tumors, Paris, France; ^4^ Oak Ridge Associated Universities, Research Triangle Park, Durham, NC, United States; ^5^ Center for Public Health and Environmental Assessment, Office of Research and Development, U.S. Environmental Protection Agency, Research Triangle Park, Durham, NC, United States; ^6^ National Centre for Asbestos Related Diseases, University of Western Australia, Perth, WA, Australia; ^7^ Institute of Environmental Medicine, Karolinska Institute, Stockholm, Sweden; ^8^ Maastricht Centre for Systems Biology (MaCSBio), Maastricht University, Maastricht, Netherlands; ^9^ Department of Medical Oncology, Inselspital, Bern University Hospital, University of Bern, Bern, Switzerland

**Keywords:** malignant pleural mesothelioma (MPM), WikiPathways, disease map, cancer, pathway modelling, BAP1

## Abstract

Malignant pleural mesothelioma (MPM) is a highly aggressive malignancy mainly triggered by exposure to asbestos and characterized by complex biology. A significant body of knowledge has been generated over the decades by the research community which has improved our understanding of the disease toward prevention, diagnostic opportunities and new treatments. Omics technologies are opening for additional levels of information and hypotheses. Given the growing complexity and technological spread of biological knowledge in MPM, there is an increasing need for an integrating tool that may allow scientists to access the information and analyze data in a simple and interactive way. We envisioned that a platform to capture this widespread and fast-growing body of knowledge in a machine-readable and simple visual format together with tools for automated large-scale data analysis could be an important support for the work of the general scientist in MPM and for the community to share, critically discuss, distribute and eventually advance scientific results. Toward this goal, with the support of experts in the field and informed by existing literature, we have developed the first version of a molecular pathway model of MPM in the biological pathway database WikiPathways. This provides a visual and interactive overview of interactions and connections between the most central genes, proteins and molecular pathways known to be involved or altered in MPM. Currently, 455 unique genes and 247 interactions are included, derived after stringent manual curation of an initial 39 literature references. The pathway model provides a directly employable research tool with links to common databases and repositories for the exploration and the analysis of omics data. The resource is publicly available in the WikiPathways database (Wikipathways : WP5087) and continues to be under development and curation by the community, enabling the scientists in MPM to actively participate in the prioritization of shared biological knowledge.

## Introduction

Malignant pleural mesothelioma (MPM) is a rare and highly aggressive cancer that affects the mesothelial cells of the pleural cavity. The main cause of MPM is asbestos exposure (1, 2). In addition, novel asbestos-like materials are rapidly emerging within innovation and technology development, and the field of toxicology requires new ways of assessing the risk of such materials to cause MPM.

Despite decades of research, the underlying causal mechanisms of MPM are still not fully understood with limited opportunities for treatments and a poor outcome (3, 4). *BAP1*, *NF2*, *CDKN2A*, *TP53*, *LATS2* or *SETD2* are a few examples of crucial genes affected in MPM which leads to well-demonstrated pathway dysregulation associated with cancer hallmarks like proliferation, angiogenesis, migration, resistance to apoptosis and others (5–7). The growing adoption of omics strategies is further expanding our level of knowledge about the cancer, opening up possibilities for new biological interpretation, clinical classification and potential treatments (6–11). At the same time, the complexity and the spread of this novel body of knowledge across multiple different databases and within specialized journals makes it increasingly challenging for the general researcher in MPM to access and employ these data. Although excellent knowledge bases already exist to collect omics and large-scale biological data (like Reactome (12), KEGG (13), cBioPortal (14, 15), PathwayCommons (16), DisGeNet (17), Comparative Toxicogenomics Database (18) and others), none of these knowledge bases is specifically MPM centered and focused on the needs and priorities of the MPM community. Further, in general, these databases do not support the direct participation of the single scientist in establishing the shared community knowledge.

We believe that creating a knowledge base platform dedicated entirely to MPM would be important for the MPM community to collect and summarize the fast-growing body of knowledge about the disease and to allow large-scale data analysis for the single scientist in MPM. We have therefore established a community portal for MPM within the WikiPathway environment, integrating recent omics information within long-standing biological knowledge about MPM, structured along the key pathways relevant for the disease. WikiPathways is a community-driven data base of biological pathways (19) that allows for a “human friendly” graphical overview of complex biology together with a machine-readable information content for data and literature collections and automated large-scale data analysis (20, 21). Based on the pathway editor PathVisio, it enables the researcher of the community to analyze and interpret their own datasets within the biological context of the pathways. At the same time the community user can actively participate and contribute to the development of the database (22).

This paper describes the development of the WikiPathways community portal dedicated to MPM, publicly available at www.wikipathways.org/instance/WP5087. Within the portal, we have linked existing knowledge pathways about MPM to other databases for (omics) data analysis, including molecular descriptors of genes, proteins and metabolites, additional molecular pathways and literature references. We zoom in on one of the highly relevant pathway sections (*BAP1*) to present an example of how such a resource may be used (23–25). We present the potential use of such a community-driven pathway-based approach together with links to tutorials that can help the general researcher in MPM to work with the knowledge base.

## Methods

### Literature Curation

A literature study was performed on peer-reviewed scientific papers reported in PubMed in July 2021 using the search terms ‘Malignant Pleural Mesothelioma’ combined with ‘genetics’, ‘molecular pathway’, or ‘signaling pathways’. Inclusion criteria were publication date after 2015, human relevance, and in the case of research papers, a focus on genetics and ‘omics approaches which was assessed through the title and abstract. Papers were reviewed for their content evidence and supporting literature. To focus particularly on MPM rather than the causative toxicological processes, papers explicitly exploring asbestos were excluded. Articles published before 2015 were referenced as well if referenced in reviews that required more detailed exploration for additional details to understand the molecular processes. Generally, all molecular entities, interactions and textual descriptions were collected and used as input to model the pathway. Of note, however, the literature curation was not intended to be a fully comprehensive systematic review but rather focused on capturing the most central and well-known mechanistic aspects of MPM, through review of a critical mass of peer-reviewed papers.

### Pathway Modelling

Based on the reported findings and descriptions in the identified papers, a molecular pathway was constructed in PathVisio (version 3.3.0) (22). Common pathway modelling practices, standard naming conventions and identifiers were implemented for all nodes describing genes, proteins, metabolites and pathways (21). Genes were included with their HGNC symbols (26) as labels and annotated with their corresponding Ensembl (27) identifiers as so-called GeneProduct nodes (black rectangles) (28). The most frequently mutated tumor suppressor genes and oncogenes were highlighted as red and yellow hexagons, respectively. Proteins were defined as such only if a single gene can have multiple different transcripts, and were annotated with UniProt identifiers (29). Metabolites were drawn as blue rectangles and annotated with their corresponding ChEBI identifiers (30). Furthermore, additional links and notes were added to the frequently mutated genes with the “comments” option in PathVisio.

Interactions between genes, proteins, complexes and metabolites were illustrated using Molecular Interaction Maps (MIM) interactions (31) to describe interactions, conversions, inhibitions, and other relevant interaction types.

To extend detailed information and avoid duplicate content in the MPM pathway, common processes described elsewhere in the WikiPathways database were referred to by the relevant pathway nodes within this pathway (green rounded rectangles) and annotated with a WikiPathways identifier.

Literature citations to papers were included in the whole pathway, specific sections, and particular interactions when appropriate. This was done using the ‘add literature reference’ option in PathVisio that created correct citations with the input of PubMed IDs.

For visual clarification about cellular localization of genes, proteins and interactions, the graphics panel of PathVisio was used to draw the cellular membrane, nucleus, mitochondria and endoplasmic reticulum.

Using the WikiPathways plugin in PathVisio, the pathway was uploaded to WikiPathways (19) and the pathway is made publicly available at www.wikipathways.org/instance/WP5087.

## Results

**Figure 1:**
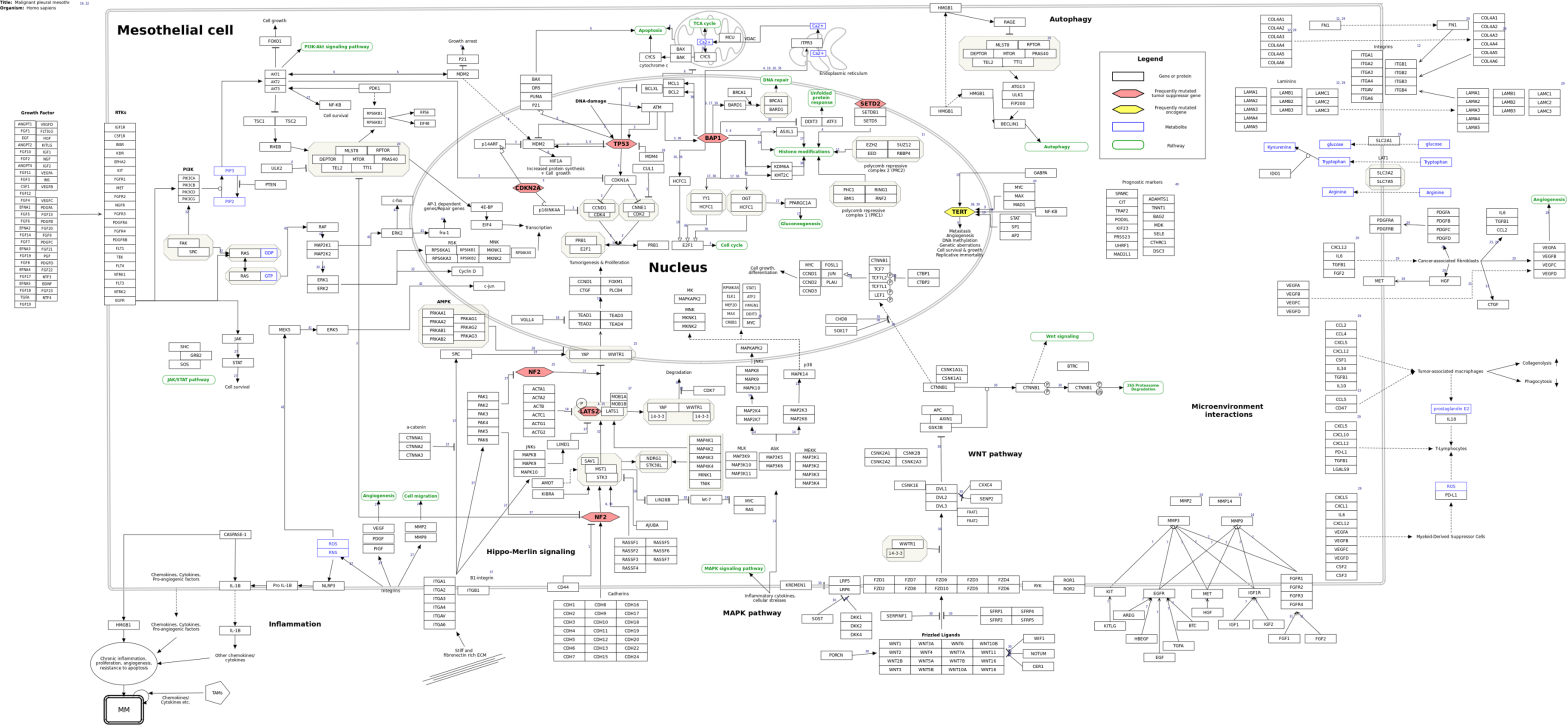
Molecular pathway of Malignant Pleural Mesothelioma from WikiPathways (wikipathways.org/instance/WP5087). Genes and proteins are visualized as black rectangles, and frequently mutated tumor-suppressor genes and oncogenes are indicated as red and yellow diamonds, respectively. Blue rectangles indicate metabolites and green rounded rectangles are links to other pathway entries in the WikiPathways database.

Based on the 26 review papers and fifteen research papers found in the literature study, a molecular pathway of MPM was constructed (**Figure 1**). The pathway is a summary of the most predominant signaling events discussed in the recent literature. In total, the pathway includes 455 genes/gene products, thirteen pathways, and fourteen metabolites, as well as 247 unique interactions between the various entities of the pathway.

From our literature curation, we found the genes *BAP1* (BRCA1 associated protein-1), *NF2* (moesin-ezrin-radixin like (MERLIN) tumor suppressor)*, LATS2* (large tumor suppressor kinase 2), *CDKN2A* (cyclin dependent kinase inhibitor 2A), *TP53* (tumor protein P53) and *SETD2* (SET domain containing 2) to be among the most frequently mutated tumor suppressors in MPM together with frequently observed mutations of the TERT (telomerase reverse transcriptase) promoter region. These genes are important components of key tumorigenic processes in MPM such as the Hippo-merlin pathway, DNA damage response, cell survival, cell cycle, and epigenetic alterations. After having established the background biological pathway information about MPM, we therefore focused our attention toward more detailed information related to these genes, which we considered to be a useful starting point to begin building our first version of the MPM molecular pathway model.

**Figure 2:**
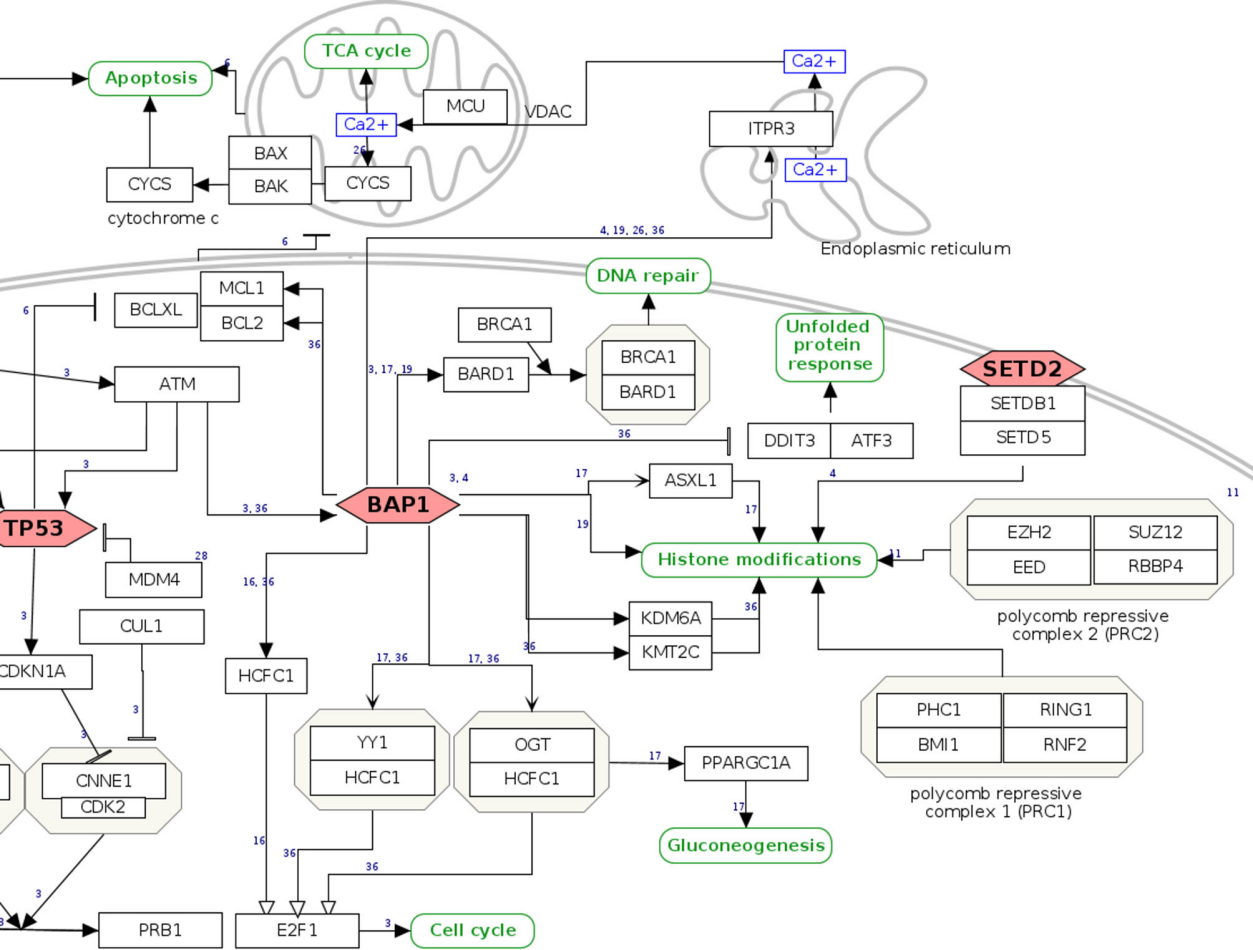
*BAP1* pathway section. Genes and proteins are visualized as black rectangles, and frequently mutated tumor-suppressor genes are drawn as red diamonds. Green rounded rectangles are links to other pathway entries in the WikiPathways database.

As a user example, we zoom in on the pathway section relating to the tumor suppressor *BAP1*, a gene with both germline and somatic inactivating mutations in MPM. *BAP1* has increasing relevance for MPM, being involved in susceptibility for the disease (24, 32), as a diagnostic (33, 34) and possibly predictive biomarker of response to treatment (35, 36). Normally, cells with inactivating mutations in *BAP1* undergo apoptosis through *RNF2*-mediated suppression of pro-survival genes *BCL2* and *MCL1*. However, in mesothelial cells, apoptosis seems not to be regulated in an *RNF2*-dependent manner, which may in part explain the tumorigenic effect associated with loss of *BAP1 (* 37 *)*. In the graphical view of the WikiPathway, the mainly nuclear localization of the BAP1 protein is directly apparent together with its function as a tumor suppressor gene, denoted by the red, hexagonal frame (**Figure 2**). A hyperlink to the Ensembl and Entrez Gene (38) databases informs about the chromosomal location and the characteristic of the gene, while for gene expression the user is directed to the RefSeq database (39) and the USSC Genome Browser (40). The function of the gene is put in context *via* the Gene Ontology knowledgebase (41, 42). Detailed Information at the protein level can be directly accessed from the UniProt database (29) and neXtProt database (43). The red, hexagonal frame of *BAP1* indicates that mutations of the gene are frequently observed in MPM according to literature. For an overview of the mutations observed in tumor samples and that could potentially be relevant for clinical interpretations, a hyperlink is provided that directs to The Cancer Genome Atlas (https://www.cancer.gov/tcga) and the OncoKB (44) data portals, together with the related references from the recent literature. Further, BAP1 acts as a deubiquitinating enzyme that regulates the activity of various targets such as ITPR3, HCFC1, BRCA1/BARD1 complex, and forming active complexes with HCFC1, YY1, OGT, and ASXL1, among others. Hence, it is involved in regulating apoptosis, proliferation, differentiation, DNA double-strand break repair, metabolic processes, and histone modifications. The network of BAP1 functional interactors and direct partners is reported with hyperlinks to the databases and portals of genes and the involved pathways.

## Discussion

We have created a pathway knowledge base platform for MPM which contextualizes the biological data available to the general researchers in MPM and permits large-scale data analysis and interpretation. The core of the platform is the initial MPM pathway model that we have derived from the curation of selected literature from the last six years. Our literature search is not an exhaustive and comprehensive overview about MPM, but a selected starting point under constant evolution and focused mainly on biomedical information. We have therefore excluded detailed search about the effects of asbestos on MPM causation and non-human studies. The MPM pathway model is drawn with PathVisio (22) and offers an intuitive visual overview of key genes and interactions in the cellular context. Each researcher can contribute to this initial model by modifying or extending the elements of the pathway. Using the drag-and-drop system of PathVisio new nodes (genes, proteins or other molecules) and interactions can be manually added or removed, reporting on the experimental or literature evidence (21). Additional information from genomics, transcriptomics, functional analysis and more can be accessed for each gene by clicking on the icon, which links to complementary databases and knowledge bases using the BridgeDb identifier mapping (45). The single user can introduce links to additional resources, if needed. A quantitative metric can be added for the nodes using color codes (e.g. gene expression data from the users own experiments or from public repositories), which also permit to combine quantitative information from different datasets (e.g. transcriptomics and proteomics quantitative data). Images of the visualization can be downloaded with different graphic formats to be used for example for publications. Beyond their visual representation, the data of the platform are annotated and published in a machine-readable way, which allows for automated data analysis. In this way the users can upload their own dataset or rely on public available resources to investigate for alterations in nodes and pathway across different conditions (e.g. disease states, treatment cohorts, gene variants and mutations, etc.), supported by statistics. This provides an important resource for the mechanistic interpretation of omics and large scale data experiments within the biomedical context of interest (46–49). The approach has been applied in several studies to investigate for example carcinogenesis (50), biomarkers (46, 51), diagnostics and therapeutics (52, 53) or to explain mechanisms of diseases (54) and the effects of toxicants (48, 53). Several plug-ins allow for the expansion and visualization of the data within their network and interaction context [e.g. Cytoscape (55, 56), CyTargetLinker (57) and STRING (58)].

Further, the machine-readability contents of WikiPathways provides a unique high level of interoperability with all major databases for genes, proteins, chemicals and probes as well as direct links to PubMed entries, PubMedCentral, and Scholia (59), and is annotated with the Pathway Ontology (60), Human Disease Ontology (61) and Cell Type Ontology (62) for improved semantic meaning and usefulness in computational approaches. These aspects make the pathway compliant to most types of data for analysis purposes, allow for a variety of computational approaches, workflows, and integrations with other tools and databases (20, 21), and align with the recently established FAIR (Findable, Accessible, Interoperable and Reusable) guiding principles for scientific data management and stewardship (63), which dictate the future of data-driven science.

The WikiPathways environment of the MPM pathway supports and fosters the direct participation of the single researcher to the scientific discussion about MPM. On one hand, the community user can directly contribute to the structure of the pathway through PathVisio, as reported above, and push the changes to update the live version on the WikiPathways website. The user can zoom in on single elements of the pathway and create subsections stored in an independent webpage linked to the main one. On the other hand, once logged in, community users can comment and start discussions through the “Discussion” tab, and stay updated about changes to the pathway by adding it to their “watch” list. Because of this interactive and visual structure, the platform is also an excellent support for education and teaching. In contrast to static knowledge in textbooks, the MPM pathway is a “living” source of updated information which undergoes continuous development and improvement as new findings and insights are published (21).

To support the users of the platform, a series of tutorials and user manuals has been created on how to perform pathway modelling and apply the elements and plug-ins of the platform (e.g. PathVisio). These users supports are accessible on the website of WikiPathways (https://www.wikipathways.org/index.php/Help:Contents). Further, the WikiPathways team provides direct assistance to the users that can also directly request for changes in the pathway. The WikiPathway team provide also a preliminary curation of the changes introduced by the single user, before updates are published in the webpage.

## Conclusion

In summary, within WikiPathways, we have provided the initial pathway model relevant for biomedical research in MPM. Different from other pathway knowledge bases, the WikiPathways model is disease-centered and focuses on the elements relevant specifically for MPM. It integrates biological knowledge about MPM with direct access to omics data and other systems biology motivated resources. It allows the researcher in MPM to perform large scale data analysis from publicly available sources and from its own research data. The platform fosters the direct participation of the community scientist in shaping and progressing our shared ongoing knowledge about MPM.

## Data Availability Statement

The datasets presented in this study can be found in online repositories. The names of the repository/repositories and accession number(s) can be found below: https://www.wikipathways.org/index.php/Pathway:WP5087.

## Author Contributions

MMa, CE, FK, FC, and PN contributed to the conception of the work. MMa and FK developed the pathway model. FC, CE, PN, HM, MMe, DJ, FE, and AN have interpreted the work and provided substantial feedback to the model development. MMa, FK, and FC drafted the manuscript. All authors have read, revised, and approved the manuscript.

## Funding

This project has received funding from the European Union’s Horizon 2020 research and innovation programme under grant agreement No 681002 (EU-ToxRisk) and under the EJP RD COFUND-EJP No 825575. This work was funded by VHP4Safety – the Virtual Human Platform for safety assessment project NWA 1292.19.272. This project has received funding from Inserm, the Marlies Schwegler Foundation, and the Swedish fund for Research without Animals (N2020-0005). This work was supported by the US Environmental Protection Agency National Program in Chemistry Safety and Sustainability, Adverse Outcome Pathway, 4.3.2: Computational modeling approaches and case study validation to identify genetic-based susceptibility factors to environmental chemical exposures.

## Conflict of Interest

The authors declare that the research was conducted in the absence of any commercial or financial relationships that could be construed as a potential conflict of interest.

## Publisher’s Note

All claims expressed in this article are solely those of the authors and do not necessarily represent those of their affiliated organizations, or those of the publisher, the editors and the reviewers. Any product that may be evaluated in this article, or claim that may be made by its manufacturer, is not guaranteed or endorsed by the publisher.
